# Effects of multidomain versus single-domain training on executive control and memory in older adults: study protocol for a randomized controlled trial

**DOI:** 10.1186/s13063-020-04293-3

**Published:** 2020-05-14

**Authors:** Soledad Ballesteros, Jennifer A. Rieker, Julia Mayas, Antonio Prieto, Pilar Toril, María Pilar Jiménez, José Manuel Reales

**Affiliations:** 1 Studies on Aging and Neurodegenerative Diseases Research Group, Universidad Nacional de Educación a Distancia, Madrid, Spain; 2 Department of Basic Psychology II, Facultad de Psicología, Universidad Nacional de Educación a Distancia, Juan del Rosal, 10, Madrid, Spain; 3 Department Methodology of Behavioral Sciences, Facultad de Psicología, Universidad Nacional de Educación a Distancia, Madrid, Spain

**Keywords:** Aging, Cognitive training, Physical exercise, Multidomain training, Executive functions, Memory functions, Randomized controlled trial

## Abstract

**Background:**

Previous research suggests that both cognitive training and physical exercise help to maintain brain health and cognitive functions that decline with age. Some studies indicate that combined interventions may produce larger effects than each intervention alone. The aim of this study is to investigate the effects of combined cognitive and physical training compared to cognitive training and physical training alone on executive control and memory functions in healthy older adults.

**Objectives:**

The main objectives of this four-arm randomized controlled trial (RCT) are: to investigate the synergetic effects of a simultaneous, group-based multidomain training program that combines cognitive video-game training with physical exercise, in comparison to those produced by cognitive training combined with physical control activity, physical training combined with cognitive control activity, or a combination of both control activities; to investigate whether event-related potential latencies of the P2 component are shorter and N2 and P3b components assessed in a memory-based task switching task are enhanced after training; and to find out whether possible enhancements persist after a 3-month period without training.

**Methods:**

In this randomized, single-blind, controlled trial, 144 participants will be randomly assigned to one of the four combinations of cognitive training and physical exercise. The cognitive component will be either video-game training (cognitive intervention, CI) or video games not specifically designed to train cognition (cognitive control, CC). The physical exercise component will either emphasize endurance, strength, and music–movement coordination (exercise intervention, EI) or stretching, toning, and relaxation (exercise control, EC).

**Discussion:**

This RCT will investigate the short and long-term effects of multidomain training, compared to cognitive training and physical training alone, on executive control and memory functions in healthy older adults, in comparison with the performance of an active control group.

**Trial registration:**

ClinicalTrials.gov, NCT03823183. Registered on 21 January 2019.

## Background

Age-related cognitive decline affects negatively the performance of daily living activities and the quality of life of many older adults. Neurocognitive frailty is the principal threat to successful aging [1, 2] as cognitive performance is central for daily life [3]. Cross-sectional studies have reported declines in a series of cognitive abilities [4–7], although these declines are less pronounced in longitudinal studies [5]. Aging is associated with a progressive decline in a wide range of cognitive abilities, such as set shifting [6], working memory [4, 6], and episodic memory [5, 7, 8]. Yet other cognitive functions which rely on previous experience, such as vocabulary and general knowledge [4, 9, 10], procedural knowledge [11], and implicit memory [12–15], are mainly preserved, not only in healthy older adults but also in those with mild cognitive impairment [16], people with Alzheimer disease [14, 17], and older adults with type 2 diabetes mellitus [18].

Cerebral aging is associated with gray and white matter reduction in several areas of the brain, including the lateral prefrontal cortex, the cerebellum, and the medial temporal lobe system including the hippocampus. In contrast, minimal decreases occur in the entorhinal and occipital cortices [19]. The prefrontal cortex organizes the incoming information and interacts with the hippocampus while performing working-memory tasks [20, 21]. Cognitive-control functions refer to the ability to adapt behavior in order to process only relevant over competing irrelevant information to attain certain goals. Neuroanatomical changes occurring in the lateral prefrontal cortex and the medial temporal lobe–hippocampus complex are associated with declines in executive functions, working memory, and episodic memory. The failure of these basic cognitive functions predicts upcoming difficulties with the performance of daily-living activities and compromises independent living [22]. However, even in advanced age, the human brain preserves a certain degree of plasticity and functional reorganization, which allows people to adapt to age-related cerebral changes in order to maintain successful task performance [23–25]. Neuroplasticity in older adults is contingent on individual behavior [26–30] and is susceptible to be modified by interventions designed to delay or prevent age-related cognitive decline [31]. Brain plasticity and its role in neural adaptations to age-related cerebral changes are also influenced by comorbidities, environmental factors, personality traits (psychosocial variables), and genetic and epigenetic factors [32]. A recent *Frontiers Research Topic* monograph focused on research conducted in the field of cognitive and brain plasticity induced by physical activity, cognitive training (computerized interventions, learning therapy, video games), and combined-intervention approaches, as well as other forms of brain stimulation that target brain activity, such as electroencephalography and neurofeedback [33]. During the last two decades, researchers have conducted a variety of intervention studies directed to promote behavioral flexibility and to enhance several cognitive processes that decline with age. Indeed, evidence for the benefits of cognitive training, video games, and physical exercise is growing rapidly, as well as research directed at gaining a better understanding of the underlying mechanisms and their translation to clinical practice [34–36].

Cognitive training is an intervention that allows structured training in a series of tasks relevant to different cognitive functions, such as executive functions, speed of processing, episodic memory, cognitive control, or attention. Among cognitive psychologists and neuroscientists there is increasing interest in exploring whether cognitive training with specially designed computerized training programs and video games of different kinds enhances cognition. Video games are electronic games that require interaction with a computer or other electronic devices with a user interface that provides visual and auditory feedback. Computerized cognitive programs and video games are currently receiving great attention in exploring the possibility of transfer to untrained tasks [37–42]. Many intervention studies based on cognitive training support the idea that training in older adults improves some aspects of cognition but not others. In recent years, several meta-analyses [43–47] have examined the effectiveness of computer-based interventions in healthy older adults. These meta-analytic studies have shown low to moderate training effects in older adults in several cognitive processes that decline with age, such as processing speed, attention, and memory. However, others [48] have reported that playing video games had little consequences on cognition. Due to different study designs (e.g., the inclusion of active or passive control groups [49, 50]) and types of training (e.g., video games of different kinds, computerized cognitive programs [51–54]), results have been heterogeneous, making it difficult to reach solid conclusions [55].

In addition, other types of training such as physical activity of different kinds are also explored as a way to improve the physical and cognitive status. The term “physical activity” includes a large number of activities related to voluntary body movements [32]. A large body of evidence supports the beneficial effects of physical activity on executive functions and memory [56–61]. Although early physical activity intervention studies, which mainly centered on cardiovascular training, showed that cardiovascular activity produced increases in hippocampal volume in older adults while improving spatial memory performance [62, 63], other types of physical exercise, such as motor fitness and coordination training, also resulted in increased hippocampal volume in healthy older adults [60]. Complex physical activities such as dancing [64–66] or the practice of martial arts [67–71] have also shown beneficial effects on cognition in older adults.

Several studies suggest that social engagement plays a key role in the maintenance of cognitive functioning and psychological well-being in older adults [32, 72, 73] (for a recent review, see [74]). In the present multidomain intervention, social engagement is not considered a source of variance, as it is not a factor manipulated in the intervention, but, rather, a design feature included to enhance cognitive and physical functioning. So, cognitive and physical training, as well as their control activities, will be performed in a social environment. In this way, the four groups will be trained in the same social conditions; that is, in small groups and in the presence of a trainer.

### Objectives and hypotheses

The main objective of this randomized controlled trial (RCT) is to investigate the synergetic effects of a group-based multidomain training program that combines cognitive video-game training with physical exercise, in comparison to those produced by cognitive training combined with physical control activity, physical training combined with cognitive control activity, or a combination of both control activities, on behavioral and electrophysiological measures of executive control (set-shifting, response inhibition, and information updating and monitoring) and memory functions (immediate and delayed visual and verbal memory). These cognitive functions, which are often compromised in later years, are essential for everyday activities. The second objective is to investigate whether event-related potential (ERP) latencies of the P2 component are shorter and N2 and P3b components assessed in a memory-based task-switching task are enhanced after training. Electrophysiology provides a very useful online measure to identify the contribution of different processing stages of executive functioning. ERPs can help us to understand the specific executive control impairments occurring with age, as well as the possible effects of the different types of intervention investigated in this RCT. To this end, the task-switching paradigm is a valid task that helps to identify the cognitive processes that most decline with ageing [75]. However, electrophysiological studies conducted to evaluate training-related effects in older adults using this task are scarce [76]. Finally, we are interested in finding out whether possible enhancements persist after a 3-month period without training.

We expect to find greater behavioral improvements in executive control and memory functions after training, larger maintenance effects, and shorter ERP latencies of the P2 component and enhanced N2 and P3b components in the multidomain training condition in comparison to both single-domain conditions. We also expect the multidomain group and both single-domain groups to outperform the active control group at the 3-month follow-up period.

In this RCT, we will use questionnaire data to verify that the groups do not differ in their levels of intrinsic motivation and engagement. At the end of the assessment session, participants will report their expectations regarding their performance in the assessment tasks using a 5-point Likert scale. Moreover, at the 1st, 8th, and 16th training session, the participants will respond to questions about motivation and engagement for each of the training video games. These factors will be examined by comparing the intervention arms to the active control condition. The engagement and motivation data will be used in secondary analyses as covariates in order to rule out these factors as sources of variation in the primary outcome variables.

## Methods

**Fig. 1:**
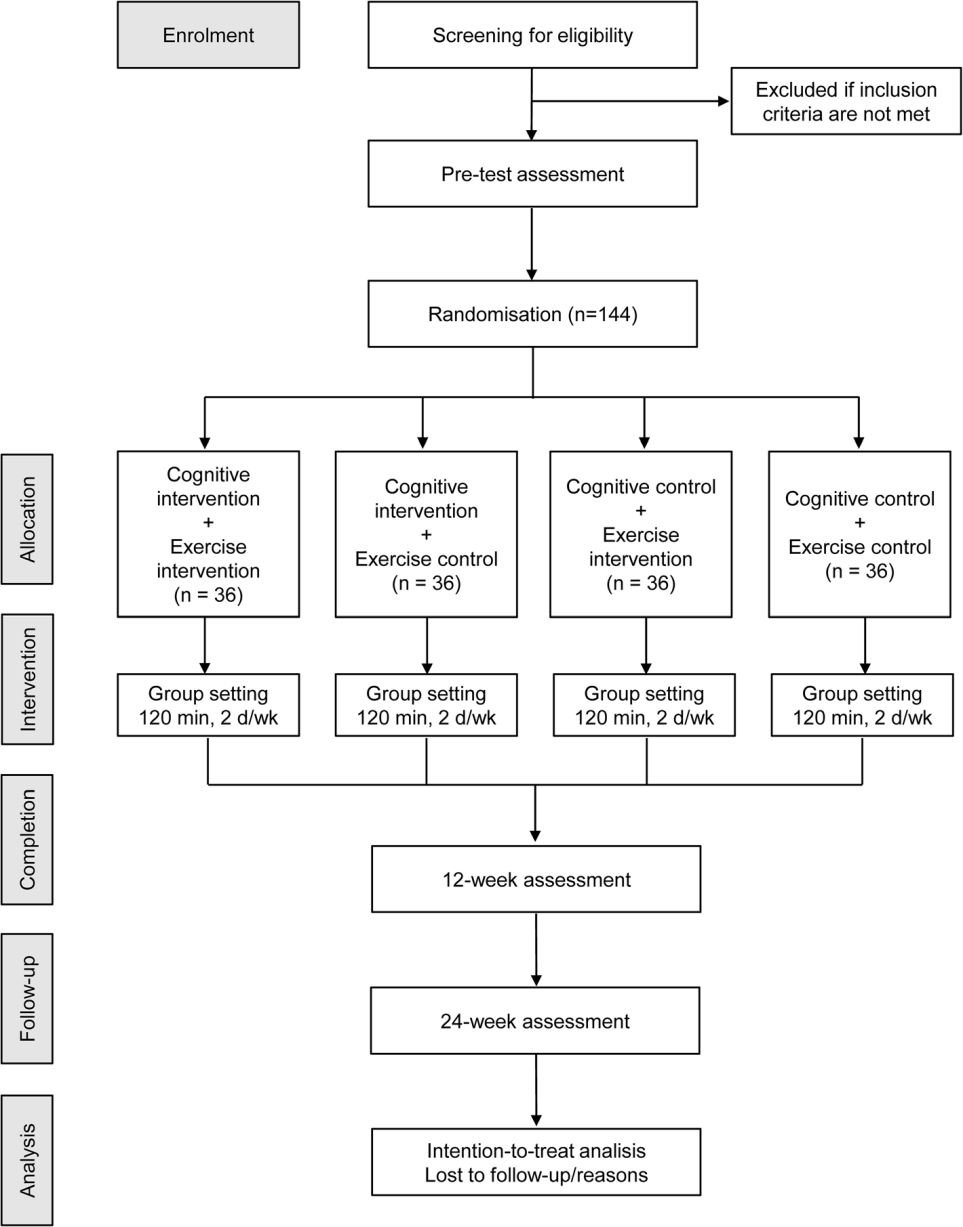
Flow chart of the study protocol. d/wk days per week.

The design is a four-arm, parallel RCT designed to investigate the effectiveness of combined cognitive and physical training versus cognitive and physical training alone but combined with a control activity, in comparison to an active control group, to promote cognitive and neurofunctional improvements in older adults. Fig. 1 shows the Consolidated Standards of Reporting Trials flow diagram corresponding to the present study.

### Study design

Participants will complete one of the four combinations of cognitive training with video games and physical exercise. The cognitive component will be either a brain-training video-game program selected from Lumosity (cognitive intervention, CI) or video games not specifically designed to train particular cognitive functions such as attention, memory, or executive control (cognitive control, CC). The physical exercise component will be either a senior-friendly adaption of BODYATTACK™ (https://www.lesmills.com/), a combination of dance, aerobic, strength, and muscular resistance (exercise intervention, EI), or its control condition comprising stretching, toning, and relaxation (exercise control, EC). The duration of sessions for all groups will be the same, and all participants will perform physical exercise and video-gaming activities in a social environment (in small groups formed by 10–12 participants) and in the presence of the trainer.

To summarize, the study will have a 4 × 3 mixed factorial design with four intervention conditions—multidomain (CI + EI), uni-domain cognitive intervention (CI + EC), uni-domain physical intervention (EI + CC), and active control (CC + EC)—assessed at three different time points (pretest, posttest, 3-month follow-up), with “Type of training” as between-subject factors and “Time” as the within-subject factor. The dependent variables will be behavioral and/or electrophysiological measures of executive functions (inhibition, shifting, working memory), memory functions (short term and long-term visual and word memory), and emotional well-being, quality of life, and motivation.

### Trial setting

This study will be conducted in Madrid (Spain) at the UNED Psychology building. The screening, pretest, posttest, and follow-up assessments will be conducted in our laboratories at the Psychology building. The training sessions of the four groups will be conducted in three waves in spaces specifically equipped and prepared for this purpose at our university site as well as at a city council facility.

### Participants

Participants will be male and female, healthy, and independently living volunteers aged between 60 and 80 years. Those who complete the baseline assessments and meet the inclusion criteria (see later) will be randomly assigned to one of the four intervention conditions. The timing of group allocation will take place between 1 and 8 weeks after baseline.

Participants may discontinue their intervention for personal or medical reasons. To minimize dropouts and improve adherence to the intervention, four face-to-face adherence reminder sessions will take place during the training program emphasizing the importance of training compliance. Furthermore, to increase participant retention and to reduce loss to follow-up, all participants will receive a personalized report of their performance and training progress at the end of the study. Participants will receive a small refund in compensation for their travelling expenses.

Even though this trial is low risk, participants might harm themselves during the practice of physical exercise. To minimize the risk of injuries, each participant will be carefully monitored during the training sessions. The interventions will be designed in collaboration with the exercise instructors, after a detailed analysis and taking into account each participant’s possible medical issues. Spontaneously reported adverse events or other unintended effects will be registered and analyzed, and if necessary the protocol will be modified to eliminate the causing element. We have signed an insurance policy in case any participant suffers harm during the physical training.

### Inclusion and exclusion criteria

Participants will have normal or corrected to normal vision and hearing, and will be free of neurological or musculoskeletal conditions, psychiatric conditions, or traumatic brain damage. They will not practice intense sports or other forms of physical exercise and will not play video games of any sort for more than 1 h per week. To determine eligibility, participants will be screened individually. Exclusion criteria will be a score of below 26 on the Mini-Mental State Examination (MMSE) [77], a score of 6 or more on the Yesavage Geriatric Depression Scale [78] (Spanish adaptation by Martínez et al. [79]), less than 20/60 vision with or without correction based on self-report, inability to complete the training activities, inability to communicate in Spanish, current plans to move to another city, and significant heart or lung disease.

### Sample size

We conducted an a priori power analysis using G*Power 3.1 [80] to calculate the appropriate sample size. Using an α value of 0.05, power of 0.80, and a medium effect size (*f* = 0.38) for video-game training [45] and physical training [81], and four groups within the *F*-test family, a total sample size of 124 is required. Considering a dropout rate of 12%, a total of 144 participants would be sufficient to detect significant main effects. According to this calculation, the adequate number of participants in each group (multidomain training, video-game training, physical activity training, and active control) is 36. According to this, we will set a sample size of 36 participants per arm, which is adequate for the experimental design. According to Montgomery et al. [82], with this number of participants the design would be underpowered to detect an interaction effect, as it would need a fourfold increase in sample size. However, given that lower interaction effects would not be clinically relevant, we decided to maintain our initial sample size estimation.

In the elaboration of this protocol, we have followed the SPIRIT 2013 explanation and elaboration guidance for reporting protocols of clinical trials [83].

### Recruitment

Participants will be recruited through organized information sessions about the project at senior programs at universities and through radio advertisements.

### Randomization and blinding

After the baseline assessments, participants will be randomly allocated to one of the four training protocols in a stratified process using the online tool Random Lists (move this to the other linehttps://www.randomlists.com/). JMR will generate the random sequence and will assign participants to interventions. At first, participants who came in couples will be randomly allocated as a unit to one of the four groups, and afterward the same procedure will be performed with the individual participants. This procedure aims to minimize dropouts due to separating couples in different groups. Participants and exercise instructors will be blinded to treatment allocation (single-blind). Data analysis will not be blinded, as it will be performed by the investigators who actively collaborate in the study. We do not envision any reason why participants should be unblinded, either during the trial or at the end of the study.

### Interventions

Participants will complete 16 training sessions of sequentially combined physical and cognitive training, or the corresponding control activities. Participants will be trained in small groups on 2 days per week for 2 h. The first 60 min of each session will be dedicated to the exercise intervention (EI) or the exercise control activity (EC), followed by 60 min of cognitive training with video games (CI) or the cognitive control activity (CC). Both CI and CC will be conducted on tablets (Brigmton BTPC 1018OC). EI and EC will be led by physical exercise instructors and accompanied by a music soundtrack.

#### Cognitive intervention

In each session, participants in the CI group will play 10 video games selected from the commercial Lumosity computerized training program (http://lumosity.com/). Lumosity provides a series of games targeting the improvement of several cognitive functions. Table 1 presents a short description of the games and their trained domains. These functions are sensitive to age-related cognitive decline and closely related to the ability to perform activities of daily living, such as driving. The participant will play the games in a predetermined sequence, for approximately 5–10 min for each game. Each participant in the CI group will have a Lumosity user account assigned. These games are adaptive—meaning that as performance improves, the difficulty increases, progressively adjusting to the participant’s performance level.

**Table 1:** Short description of the video games for the cognitive intervention

Game name	Trained function	Description
Train of Thought	Divided attention	The player directs trains to their matching station
Assist Ants	Divided attention	The player prevents collisions by placing obstacles in their paths
Trouble Brewing	Divided attention	The player simultaneously serves orders to different customers
Playing Koi	Divided attention	The player keeps track of which fish has already been fed, in a square of randomly appearing fishes
Memory Serves	Working memory and divided attention	The player matches different pieces of luggage to their corresponding owners
Disillusion	Flexibility	The game consists of matching tiles with different shapes, colors, or symbols
Ebb and Flow	Flexibility	The player swipes in the direction to which the leaves are moving or pointing
Master Piece	Spatial reasoning	The player reorientates a shape so that it fills a hollow section
Speed Pack	Visualization	The player has to fit the last item into an already filled suitcase
Highway Hazards	Information processing	The player dodges obstacles in a race through a virtual desert

#### Physical intervention

The exercise intervention will consist of BODYATTACK™, which is a registered trademark of moderate to high-intensity training that combines aerobic exercises with strength and balance exercises. During the exercise protocol, participants will train at 65–80% of their maximum heart rate. The training sessions are predetermined by the distributer and comprise standardized movements, exercises, and music soundtracks (see Table 2). Exercises include large plyometric movements and more controlled movements, and train equally upper and lower body muscles with dynamic movement coordination. The sequence of exercises is as follows: 10-min warm-up, 35-min main phase (with active recovery between intervals), and 10-min cool-down.

**Table 2:** Description of the exercise intervention

Activity	Trained function	Description
Adaptation of “BODYATTACK™”	Cardiovascular fitness	Aerobic exercises
	Endurance	Strength moves
	Coordination	Movements to music soundtrack
	Balance	Stabilization exercises
	Flexibility	Stretching

#### Cognitive control activity

The cognitive control component will exclusively involve language-specific processes and crystalized knowledge (see Table 3). These domains are preserved with age, and even though an implication of executive functioning cannot be ruled out, this is clearly not the main active component. The cognitive control games are available within the gaming service Google Play Games, which mimics cognitive training platforms. This will create the impression of receiving an intervention, thereby reducing expectation biases. Participants will play 10–15 min each game in a predetermined sequence.

**Table 3:** Short description of the video games for the cognitive control condition

Game name	Trained function	Description
Hangman	Lexical access	The player guesses a word by suggesting letters within a certain number of guesses
Grammar	Lexical access	The player chooses the correct spelling of a word within three possibilities
Definitions	Semantics and lexical access	The player chooses the correct word according to a given definition
Word search	Lexical access	The player identifies words hidden in a grid filled with letters
Crossword	Semantics and lexical access	The player constructs words by solving clues
Synonyms and antonyms	Semantics and lexical access	The player produces a word with a similar or opposite meaning of a given word
Trivia quiz	Crystalized knowledge	The player answers to questions of general knowledge

#### Physical control activity

The physical control activity will consist of BODYBALANCE™ (https://www.lesmills.com/), which is a music-guided exercise that combines Tai Chi, Yoga, and Pilates exercises. The sequence of exercises of each session is as follows: 10-min warm-up with Tai Chi exercises; 35-min main phase with Yoga and Pilates exercises with a focus on breathing, stretching, balance, and strengthening of abdominal muscles; and 10-min cool-down with meditation and relaxation. The physical activity intervention and the physical control activity are briefly described in Table 4.

**Table 4:** Description of the activities of the exercise control condition

Game name	Trained function	Description
Adaption of “BODYBALANCE™”	Flexibility	Stretching
	Relaxation	Respiratory exercises

### General procedure

After baseline, participants who meet the inclusion criteria will be randomly assigned to one of the four groups. The active control group was introduced in the design to control for placebo effects [84]. The main question is whether the multidomain group will outperform the single-domain groups at posttest, and whether these groups will outperform the active control group in a series of cognitive-control and memory tasks (see below). We focused on these cognitive domains because they deteriorate with age and are critical for independent living.

All methodological designs of primary and secondary outcomes are constructed using the rules of counterbalance and stimulus rotation. Response keys will be counterbalanced across conditions. The computerized tasks have been programmed with E-Prime 2.0 (Psychological Software Tools Inc.). Continuous EEG activity will be recorded in our laboratories with thin electrodes from 40 scalp sites using NuAmps amplifiers while participants perform the task-switching task.

### Outcome measures

**Fig. 2:**
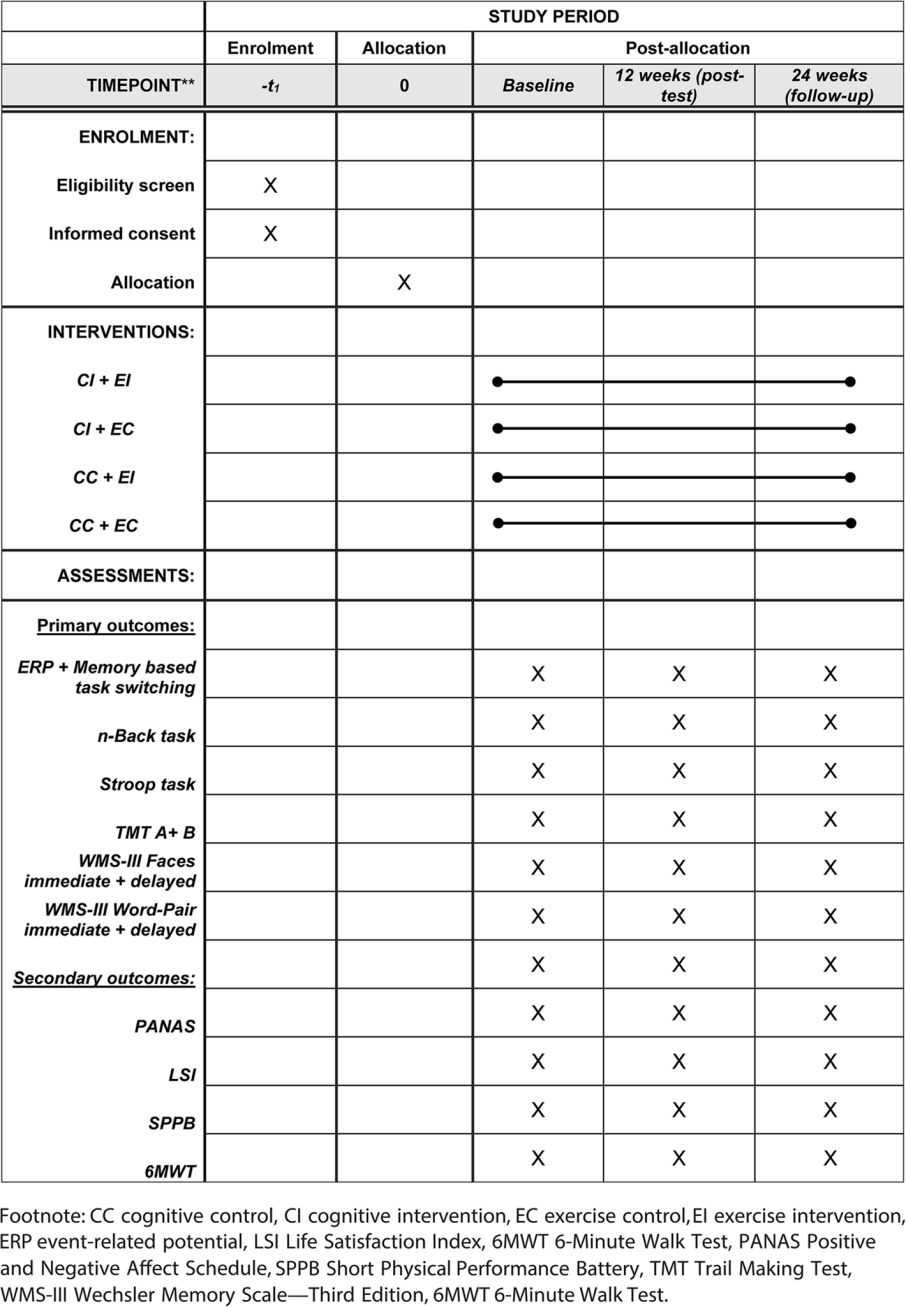
Standard Protocol Items: Recommendations for Interventional Trials (SPIRIT 2013) diagram illustrating the schedule of enrolment, post allocation, and close-out for all assessments. CC cognitive control, CI cognitive intervention, EC exercise control, EI exercise intervention, ERP event-related potential, LSI Life Satisfaction Index, 6MWT 6-Minute Walk Test, PANAS Positive and Negative Affect Schedule, SPPB Short Physical Performance Battery, TMT Trail Making Test, WMS-III Wechsler Memory Scale—Third Edition

Each group will be assessed at three time points. Possible improvement will be assessed at posttest (12 weeks) and follow-up (24 weeks) using baseline (week 0) outcomes as a reference point. A schematic diagram of the time schedule of data collection for all outcome measures is shown in Fig. 2 (see also Additional file 1: SPIRIT checklist).

To report the primary and secondary outcomes, we will follow the outcome definition proposed by Saldanha et al. [85] and Zarin et al. [86] that includes the domain, the specific measurement, the specific metric, the method of aggregation, and the time points that will be used for analysis.

### Primary outcomes: training effects on cognitive functions

#### Set-shifting

##### Memory-based task switching

Executive functions will be assessed with a memory-based task-switching paradigm. In this task [76, 87], digits from 1 to 9 (excluding number 5) are presented in white on a black background on the computer screen. A cue indicating the relevant task is presented simultaneously with the digit below the fixation point. The cue “NUM” indicates a numerical task (smaller or greater than 5), “PAR” the parity task (odd vs. even), and “TAM” (a diminutive for Spanish “tamaño” (size) font) the font-size task (small vs*.* large). Each stimulus is presented in small (7 mm × 10 mm) and large (12 mm × 18 mm) size. Participants will perform three single and two mixed blocks. In the single blocks, they have to process digits according to the one-task rule (i.e., numerical, parity, or font-size task only). In the memory-based mixed blocks, participants have to switch between different tasks within the block. In the cue block they are instructed to switch the rule after every three trials in the following order “NUM–NUM–NUM–PAR–PAR–PAR–TAM–TAM–TAM”, while a cue is presented in every trial simultaneously with the digit. In the memory block, participants are instructed to switch the rule after every three trials in the same order, while “XXX” instead of a cue is presented; that is, participants have to keep track of the trial sequence in their working memory. When three consecutive errors are made, or no response is given, cues are presented on three consecutive trials to help participants to find the track. Single blocks consist of 35 trials each, and two mixed blocks consisting of switch and no-switch trials: a cued block (90 trials) and a memory block (90 trials). The mixed blocks are equal with respect to the stimulus type, response type, and frequency of task switch (33.3%). The stimulus–response mapping of the three tasks is overlapping; that is, responses according to “smaller than 5”, “even”, and “small size” are assigned to the left key and “larger than 5”, “odd”, and “large size” to the right key. The assignment will be counterbalanced across participants. The outcomes of interest are mean reaction times (RTs) between groups corresponding to correct trials at pretest, posttest, and follow-up time points. The specific metric will be the change from baseline.

#### Processing speed and flexibility

##### Trail Making Test (TMT)

The TMT is a neuropsychological test of visual attention and task switching. The test comprises two parts (A and B). Each part consists of 25 circles distributed over a sheet of paper. In Part A, the circles are numbered 1–25, and the participant draws lines to connect the numbers in ascending order. In Part B, the circles include both numbers (1–13) and letters (A–L); the task consists of connecting the circles in an ascending pattern, but with the added task of alternating between the numbers and letters (i.e., 1–A–2–B–3–C, etc.). The total times in seconds for Parts A and B represent the TMT-A and TMT-B direct scores. Scores of TMT-A account for perceptual speed, whereas the B–A difference score is an indicator of task-switching abilities. The outcomes of interest are the mean time scores of the difference score, TMT-B minus TMT-A, to assess task switching between groups at pretest, posttest, and follow-up time points. The specific metric will be the change from baseline.

#### Working memory

The *N*-back task is a continuous performance task to assess maintenance and updating of information in working memory. This task has been used with older adults [88–90]. Participants are presented with a sequence of stimuli (consonant letters), and indicate whether the last stimulus matches the one presented “*n*” trials back by pressing one of two keys (one for “yes” or another for “no”). We used a three level *N*-back task. In the 0-back condition, the letter X is the target. We include the 0-back condition as an index of perceptual-motor speed to control for the role of speed of processing in working memory performance. In the 1-back condition, participants have to remember the stimulus presented just before the current stimulus; in the 2-back level, they have to remember the stimulus presented two positions before. Each participant first performs a practice block of 17 trials at each level, followed by the experimental trials. Each level contains three blocks of 27 trials (81 trials per level), yielding a total of 243 trials. Each block of 27 trials consists of 17 “nontargets” (“no” response) and 10 “targets” (“yes” response). The outcomes of interest are the mean accuracy between groups as assessed by Hits–False alarms. The specific metric will be the change from baseline (pretest) to posttest and follow-up.

#### Inhibitory control

The Stroop interference effect reflects the extra time needed to resolve the conflict generated by an automatically processed irrelevant dimension. The Stroop task assesses response inhibition. We use the computerized Color–Word version of the Stroop task [42] with two different conditions: in the congruent condition, color name words match with the ink color; while in the incongruent condition, color names are printed in an incompatible ink color. In both conditions, participants are instructed to name the color of the ink as soon as possible. Longer response latencies and higher error rates on incongruent trials (when the color of the letters conflicts with the word) compared to congruent trials (when color and word match) constitute the Stroop effect. The Stroop effect correlates negatively with the efficiency of inhibitory control. The Stroop task contains 18 practice trials and two experimental blocks of 126 trials each, with a proportion of incongruent trials of 66%. Responses are assigned to the keys “v”, “b” and “n”, and the stimulus–response mapping is counterbalanced across participants. The dependent variable is the mean RT corresponding to the congruent and incongruent correct trials of the groups at pretest, posttest, and follow-up. The specific metric will be the change from pretest in the computerized version of the Stroop task to assess response inhibition.

#### Immediate and differed visual and verbal memory

##### Wechsler Memory Scale—Third Edition (WMS–III) Faces

The WMS-III Faces subtest [91] (The Psychological Corporation, 1997) uses a recognition paradigm to assess immediate and delayed visual memory. In Faces I, participants are presented with 24 target faces at a speed of 2 s per picture. Then, they are shown 48 faces (24 targets and 24 distractors) and are asked to identify the target faces by responding either “yes” or “no” to each face. Participants are prompted to keep the target faces in mind. In Faces II, participants are shown 48 faces (24 targets and 24 distractors) after a 30-min delay and are asked to identify the target faces. The Hits–False alarms mean between groups at pretest, posttest, and follow-up assessments will be the outcome of interest. The specific metric will be the change from pretest to posttest and follow-up.

##### Wechsler Memory Scale—Third Edition (WMS–III) Word-Pair List

The WMS-III Word-Pair subtest assesses immediate and delayed verbal memory. In this test, four trials of eight unrelated word pairs are presented at a rate of 3 s per pair. In the immediate recall condition, after the presentation of the four lists, the first word of each pair is read to the participant, who has to provide the associated word of the pair. After a delay of approximately 25–35 min, the same procedure is repeated, and the participant provides the second word of each pair. Finally, a recognition task is administered where 24 word pairs are presented and the participant is asked to identify the pair as either “new” or “old”. The Hits–False alarms mean between groups at pretest, posttest, and follow-up assessments will be the outcome of interest. The specific metric will be the change from pretest to posttest and follow-up.

#### Electrophysiological measures

##### Electroencephalograph acquisition

While performing the experimental memory-based switching task, continuous electroencephalograph (EEG) activity will be recorded using a NuAmps amplifier (Neuroscan Inc.) inside a soundproof, electromagnetically shielded room. We will use a 34-channel elasticized Quik-Cap with Ag/AgCl sintered electrodes (American Medical EEG Association, 1991). To control ocular artifacts, vertical and horizontal electrooculograms will be recorded in two bipolar channels. Eye blinks and vertical eye movements will be monitored via electrodes located below and on the supraorbital ridge of the left eye. Horizontal artifacts will be monitored via electrodes on the outer canthus of each eye. Linked mastoids (A1, A2) will be used as a reference, and participants will be grounded to the AFz electrode. All data will be digitized using a NuAmps amplifier in continuous recording mode. The sampling rate will be 1000 Hz, and all channels will be online bandpass filtered (0.1–140 Hz) and notch filtered (50 Hz) to eliminate power line artifacts. Continuous data will be filtered offline using a digital Butterworth filter (0.1–40 Hz; 12 dB per octave roll-off), an infinite impulse response filter that achieves a given filtering characteristic. After filtering, data will be separated into baseline-corrected and nonoverlapping epochs time-locked to the target onset. Epochs containing high amplitude/frequency and muscle or other irregular artifacts will be removed by visual inspection. Only artifact-free epochs from correct trials will be selected for averaging. The existence of blinks and other ocular movements will not be a criterion for epoch rejection. This kind of artifact will be eliminated using Independent Component Analysis (ICA) [92–98]. After submitting EEG data to ICA decomposition, artifactual components will be removed by inspection of their activity, scalp topography, and spectral power. The length of the epoch in the target-locked ERP will be 1100 ms, and 600 ms in the response-locked ERP. We focus on P2, N2, and P2b. Analyses will be centered on the posttarget and postresponse ERPs at the midline electrodes located at the frontal, central, and parietal lobes (Fz, Cz, and Pz) where the components of interest are usually maximum. P2 is a positive ERP component associated with retrieval of stimulus–response sets that will be measured between 150 and 300 ms. N2 will be measured at the most negative pick between 150 and 400 ms after target onset. P3b will be measured in the time window of 300–600 ms after target onset. This wave is associated with context updating and working memory (see [75, 76, 87]).

### Secondary outcomes

#### Assessment of emotional and affective well-being

##### The Positive and Negative Affect Schedule (PANAS)

The PANAS [99] is a self-report questionnaire designed to assess the affective state. It consists of two 10-item scales to measure both positive and negative affect. Positive affect reflects the point to which a person feels enthusiastic, active, and alert, with energy and rewarding participation. Negative affect represents a general dimension of subjective distress and unpleasant participation that includes a variety of aversive states, such as disgust, anger, guilt, fear, and nervousness. Participants in the PANAS respond to a 20-item test using a 5-point scale that ranges from very slightly or not at all (1) to extremely (5). We use the Spanish version [100] that provides good consistency and reliability indexes, and also confirms the original two factors of the questionnaire. The reliability (Cronbach’s α) and validity, both convergent and discriminant, have also been corroborated in the elderly Spanish population [101].

The outcomes of interest are the mean score per group of positive and negative affect assessed with the PANAS questionnaire at three time points: pretest, posttest, and follow-up. The specific metric will be the change from baseline.

##### The Life Satisfaction Index (LSI)

The LSI [102] is a 20-item self-report questionnaire to measure psychological well-being in older adults. The instrument consists of five subscales, including zest for life (four items), resolution and fortitude (five items), congruence between desired and achieved goals (three items), positive self-concepts (three items), and mood tone. Respondents express their agreement or disagreement with the statements based on a 3-point Likert scale (agree = 2 points; disagree = 1 point; and “don’t know” = 0 points). The higher the overall score, the higher the individual’s life satisfaction.

The outcome of interest is the mean score per group of the individual’s life satisfaction assessed with the LSI questionnaire at three time points: pretest, posttest, and follow-up. The specific metric will be the change from baseline.

#### Assessment of physical condition

##### The Short Physical Performance Battery (SPPB)

The SPPB [103] measures functional status and physical performance. First described in 1994, it is a composite measure assessing walking speed, standing balance, and sit-to-stand performance. The SPPB is calculated from three components: the ability to stand for up to 10 s with feet positioned in three ways (together side by side, semi-tandem, and tandem); time to complete a 3-m or 4-m walk; and time to rise from a chair five times.

Lower-extremity physical performance is assessed in the study with a composite measure of walking speed, standing balance, and sit-to-stand performance. The outcome will be the performance mean in the battery of the groups at three time points: pretest, posttest, and follow-up. The specific metric will be the mean change from pretest to the other time points.

##### The 6-Minute Walk Test (6MWT)

The 6MWT [104] is commonly used to assess exercise capacity. The participant walks for 6 min as fast as possible. The primary outcome is the distance completed. The test is administered in accordance with the protocol endorsed by the ATS [105]. The test is performed on a straight 30-m corridor and all participants receive standardized scripted instructions and scripted phrases of encouragement each minute during the test. Besides the distance, monitored parameters are changes in oxygen saturation (SpO_2_), and pretest and posttest dyspnea and fatigue using the Borg scale [106].

Functional capacity is assessed in the study with the 6MWT. The outcome measure will be the mean absolute value in the test obtained by the groups at three time points: pretest, posttest, and follow-up. The specific metric will be the mean change from pretest to the other time points of the study.

### Statistical analysis

All data from participants with complete baseline assessment and who attended at least one training session will enter into a primary intention-to-treat analysis. For the secondary per-protocol analysis, only the data of participants with a complete cognitive assessment and an attendance rate ≥ 70% will be considered.

Executive functions (set shifting, maintenance, inhibitory control) and memory functions (short-term, visual, and verbal immediate and delayed memory) will be assessed at pretest, posttest, and follow-up. The statistical analysis corresponding to the behavioral results will be carried out with the SPSS statistical package for Windows (SPSS 25.0; IBM Corporation). Results will be considered significant at *p* < 0.05, with Bonferroni-corrected post hoc tests performed as appropriate. We will explore the missing data to ascertain their pattern and will apply an adequate technique of multiple imputation. Repeated ANOVA measures will be conducted with four groups (multidomain training, cognitive training, exercise training, active control) at three time points (pretest, posttest, follow-up) to test the primary hypothesis (i.e., differences in efficacy between interventions compared to the active control condition). Repeated ANOVA measures will also be performed to determine the effect of the interventions on secondary outcomes. To evaluate the effect size of the combined multimodal group versus each individual intervention group and the control arm, we will use multimodal regression with an interaction term. Electrophysiological data will be analyzed with CURRY 8.0.2, the EEGLAB toolbox [92], and the ERPLAB plugin for EEGLAB [98].

### Data monitoring committee and data management

Personal information about participants obtained during the individual interviews as well as performance data and all study-related information will be coded in a database and stored securely at the study site to maintain participant confidentiality. A coded identification (ID) number to maintain participant confidentiality will identify all data collection and administrative forms. The electronic data will be stored securely on a university computer and a high-drive disc (HDD) that are password protected. Paper copies as well as HDDs will be securely stored in a locked cabinet at the study site. All forms, lists, appointment records, consent forms, and any other listings that link participant ID numbers to other identification information will be stored in a separate, locked file in a limited access area. Only the members of the researcher team directly involved in data collection, maintenance, and management will have access to the data set.

The data monitoring committee (DMC) will be composed of JAR and JMR, who will regularly check on the correctness of data collection and encoding and its correspondence with the entrances in the laboratory diary. The DMC will be responsible for securing the data on a weekly basis on the aforementioned devices. The data will be stored securely in our laboratory for 5 years.

We have not planned to conduct subgroups of interim analyses.

### Steering committee

The steering committee will meet at least on a quarterly basis to monitor the trial processes, independently of the funding organization. The committee will check compliance with the assessment and training protocols and the timelines, and will oversee and manage the trial. Its members, who form an active part of the research group, are SB and JMR. They will verify trial processes, such as participant enrollment, informed consent, eligibility, allocation of participants to groups, and adherence to trial interventions.

### Dissemination plans

After completion of the trial, the results will be presented at international and national conferences and will be published in appropriate scientific journals. We will also deliver the results to the participants.

## Discussion

We investigate the potential for cognitive training and physical exercise to prevent or minimize the negative effects occurring with aging. This clinical trial examines the efficacy of a combined intervention on moderate cognitive decline as well as affective well-being and physical condition in healthy older adults. This multimodal intervention study will contribute to the increasing body of literature investigating ways to promote brain plasticity and maintain healthy and active aging.

To summarize, cognitive decline and physical decline have negative effects in older adults and impact negatively on society due to the increasing number of older adults that will suffer cognitive decline and neurodegenerative diseases in the next decades. Finding effective ways to prevent the negative impact of declining cognition would have a key effect on the current limited social and health care resources.

## Trial status

This clinical trial was registered at the National Institute of Health (NIH) with the Clinicaltrials.gov identifier NCT03823183 (https://register.clinicaltrials.gov/ClinicalTrials.gov) on 21 January 2019. The protocol version number is number 1 (January 2019). Recruitment started in February 2019 and is expected to be completed in February 2020. Once the trial is completed, results will be reported according to the Consolidated Standards of Reporting Trials (CONSORT) guidelines. The trial is active and ongoing. We expect to have the final results by the middle of 2021.

## Supplementary information

**Additional file 1.** Standard Protocol Items: Recommendations for Interventional Trials (SPIRIT) checklist.

**Additional file 2.** Table of items found in the WHO trial registry data.

## Data Availability

Access to the protocol and the dataset may be provided upon request to the authors.
