# Supplementary material for: Effects of multidomain versus single-domain training on executive control and memory in older adults: study protocol for a randomized controlled trial
Source: Trials. 2020 May 14;21:404. doi: 10.1186/s13063-020-04293-3 (PMC7222523; doi:10.1186/s13063-020-04293-3)
Supplement: Supplementary file 2 — Additional file 2. Table of items found in the WHO trial registry data. [file 13063_2020_4293_MOESM2_ESM.docx]

| **Table S1.** WHO TRIAL REGISTRY DATA SET |
| --- |
| 1. **Primary Registry and Trial Identifying Number** |
| Clinicaltrials.gov ID: NCT03823183; https://register.clinicaltrials.gov/ |
| 1. **Date of Registration in Primary Registry** |
| 21 January 2019 |
| 1. **Secondary Identifying Numbers** |
| # PSI2016-80377-R |
| 1. **Source(s) of Monetary or Material Support** |
| Public |
| 1. **Primary Sponsor** |
| Spanish Ministry of Economy and Competitiveness – Government of Spain |
| 1. **Secondary Sponsor(s)** |
| N/A |
| 1. **Contact for Public Queries** |
| Soledad Ballesteros  Studies on Aging and Neurodegenerative Diseases Research Group  Department of Basic Psychology II  Universidad Nacional de Educación a Distancia  Juan del Rosal, 10  Madrid (Spain)  Phone: 34 91398 62 27  Email: mballesteros@psi.uned.es |
| 1. **Contact for Scientific Queries** |
| Soledad Ballesteros  Studies on Aging and Neurodegenerative Diseases Research Group  Department of Basic Psychology II  Universidad Nacional de Educación a Distancia  Juan del Rosal, 10  Madrid (Spain)  Phone: 34 91398 62 27  Email: mballesteros@psi.uned.es |
| 1. **Public Title** |
| Effects of multi-domain versus single-domain training on executive control and memory in older adults: Study protocol for a randomized controlled trial |
| 1. **Scientific Title** |
| Effects of multi-domain versus single-domain training on executive control and memory in older adults: Study protocol for a randomized controlled trial |
| 1. **Countries of Recruitment** |
| Spain |
| 1. **Health Condition(s) or Problem(s) Studied** |
| Prevention of age-related cognitive decline |
| 1. **13. Intervention(s)** |
| Participants will complete 16 training sessions of sequentially combined physical and cognitive training, or its corresponding control activities. Participants will be trained in small groups 2 days/week for two hours. The first 60 min of each session will be dedicated to the exercise intervention (EI) or the exercise control activity (EC), followed by 60 min of cognitive training with video games (CI) or the cognitive control activity (CC). Both, CI and CC will be conducted on tablets (Brigmton BTPC 1018OC). EI and EC will be led by physical exercise instructors and accompanied by a music soundtrack.  **Cognitive intervention**  The cognitive intervention consists of 10 video games selected from the *Lumosity* computerized training program (<http://lumosity.com/>). The games are designed to enhance flexibility, working memory, and processing speed. The participant will play the games in a pre-determined sequence, for approximately 5 to 10 minutes each game. Each participant in the CI group will have a Lumosity user account assigned. These games are adaptive meaning that as performance improves, difficulty increases progressively adjusting to the participant´s performance level.  **Physical intervention**  The exercise intervention will consist of BODYATTACK ™, which is a registered trademark of moderate to high intensity training that combines aerobic exercises with strength and balance exercises. During the exercise protocol participants will train at 65 to 80% of their maximum heart rate. The training sessions are predetermined by the distributer and comprise standardized movements, exercises and music soundtracks. Exercises include large plyometric movements and more controlled movements, and train equally upper and lower body muscles with a dynamic movement coordination. The sequence of exercises is as follows: 10-minute warm-up, 35-minute main phase (with active recovery between intervals), and 10-minute cool-down.  **Cognitive control activity**  The cognitive control activity consists of 6 video games that exclusively involve language-specific processes and crystalized knowledge. The cognitive control games are available within the gaming service Google Play Games, which mimics cognitive training platforms. This will create the impression of receiving an intervention, reducing thereby expectation biases. Participants will play 10 to 15 minutes each game in a pre-determined sequence.  **Physical control activity**  The physical control activity will consist of BODYBALANCE ™ https://www.lesmills.com/, which is a music-guided exercise that combines Tai Chi, Yoga, and Pilates exercises. The sequence of exercises of each session is as follows: 10-minutes warm-up with Tai Chi exercises, 35-minutes main phase with Yoga and Pilates exercises with focus on breathing, stretching, balance, and strengthening of abdominal muscles, and 10-minutes cool-down with meditation and relaxation. |
| 1. **Key Inclusion and Exclusion Criteria** |
| Participants will have normal or corrected to normal vision and hearing, and will be free of neurological or musculoskeletal conditions, psychiatric conditions, or traumatic brain damage. They will not practice intense sports or other forms of physical exercise and will not play video games of any sort for more than an hour a week. To determine eligibility, participants will be screened individually. Exclusion criteria will be a score of below 26 on the Mini-Mental State Examination (MMSE), a score of 6 or more on the Yesavage Geriatric Depression Scale, less than 20/60 vision with or without correction based on self-report, inability to complete the training activities, inability to communicate in Spanish, current plans to move to another city, significant heart or lung disease. |
| 1. **Study Type** |
| The design is a four-arm parallel RCT with pre, post, and 3-month follow-up assessments. The aim of the trial is to investigate the effectiveness of combined cognitive and physical training versus cognitive and physical training alone but combined with a control activity, in comparison to an active control group, to promote cognitive and neurofunctional improvements in older adults. Participants and exercise instructors will be masked (single-blind), and treatment allocation will be generated automatically with an online random list generator. |
| 1. **Date of First Enrollment** |
| Enrolment started in January 2019 |
| 1. **Sample Size** |
| N = 144 (36 per arm) |
| 1. **Recruitment Status** |
| Recruitment and enrolment are in course. |
| 1. **Primary Outcome(s)** |
| **Outcome: Set-shifting**  Metric/method of measurement: Memory-based task-switching  Timepoints: at pretest, posttest, and 3-month follow-up  **Outcome: Inhibitory control**  Metric/method of measurement: Stroop task  Timepoints: at pretest, posttest, and 3-month follow-up  **Outcome: Working memory**  Metric/method of measurement: *N*-Back  Timepoints: at pretest, posttest, and 3-month follow-up  **Outcome: Processing speed and flexibility**  Metric/method of measurement: Trail Making Test (TMT)  Timepoints: at pretest, posttest, and 3-month follow-up  **Outcome: Immediate and differed visual memory**  Metric/method of measurement: Wechsler Memory Scale–Third Edition (WMS–III) Faces I and II  Timepoints: at pretest, posttest, and 3-month follow-up  **Outcome: Immediate and differed verbal memory**  Metric/method of measurement: Wechsler Memory Scale–Third Edition (WMS–III) Word-Pair List I and II  Timepoints: at pretest, posttest, and 3-month follow-up  **Outcome: Cerebral functionality during memory-based task switching**  Metric/method of measurement: Event-Related Potential (ERPs) recordings while performing a memory-based task switching task. Analyses will be centered at the midline electrodes (Cz and Pz) on components P2 (retrieval of stimulus-response sets), N2 (stimulus selection of task-relevant information and inhibitory control), and P3b (context updating and working memory).  Timepoints: at pretest, posttest, and 3-month follow-up |
| 1. **Key Secondary Outcomes** |
| **Outcome: Affective well-being**  Metric/method of measurement: Positive and Negative Affect Schedule (PANAS)  Timepoints: at pretest, posttest, and 3-month follow-up  **Outcome: Emotional well-being**  Metric/method of measurement: Life Satisfaction Index (LSI).  Timepoints: at pretest, postest, and 3-month follow-up  **Outcome:** **Physical exercise capacity**  Metric/method of measurement: 6-min Walk Test (6MWT)  Timepoints: at pretest, posttest, and 3-month follow-up  **Outcome: Lower extremity functioning**  Metric/method of measurement: Short Physical Performance Battery (SPPB)  Timepoints: at pretest, posttest, and 3-month follow-up |
| 1. **Ethics Review** |
| The UNED Research Ethics Committee approved the study on November 12, 2018 |
| 1. **Completion date** |
| Last subject: October 2020 |
| 1. **Summary Results** |
| 1. Date of posting of results summaries   The training phases and assessments are split into three waves with identical training and assessment protocols. For this reason, no results summaries will be posted before the final results are available by the end of 2020.   1. Date of the first journal publication of results   N/A   1. URL hyperlink(s) related to results and publications   N/A   1. Baseline characteristics   Age, sex, education and academic level, foreign language knowledge and level, leisure activities (sports, music, social live), medical information, lower extremity functioning, physical exercise capacity, emotional well-being, affective well-being, executive functions (working memory, inhibition, flexibility), immediate and differed visual and verbal memory, cerebral functionality.   1. Participant flow   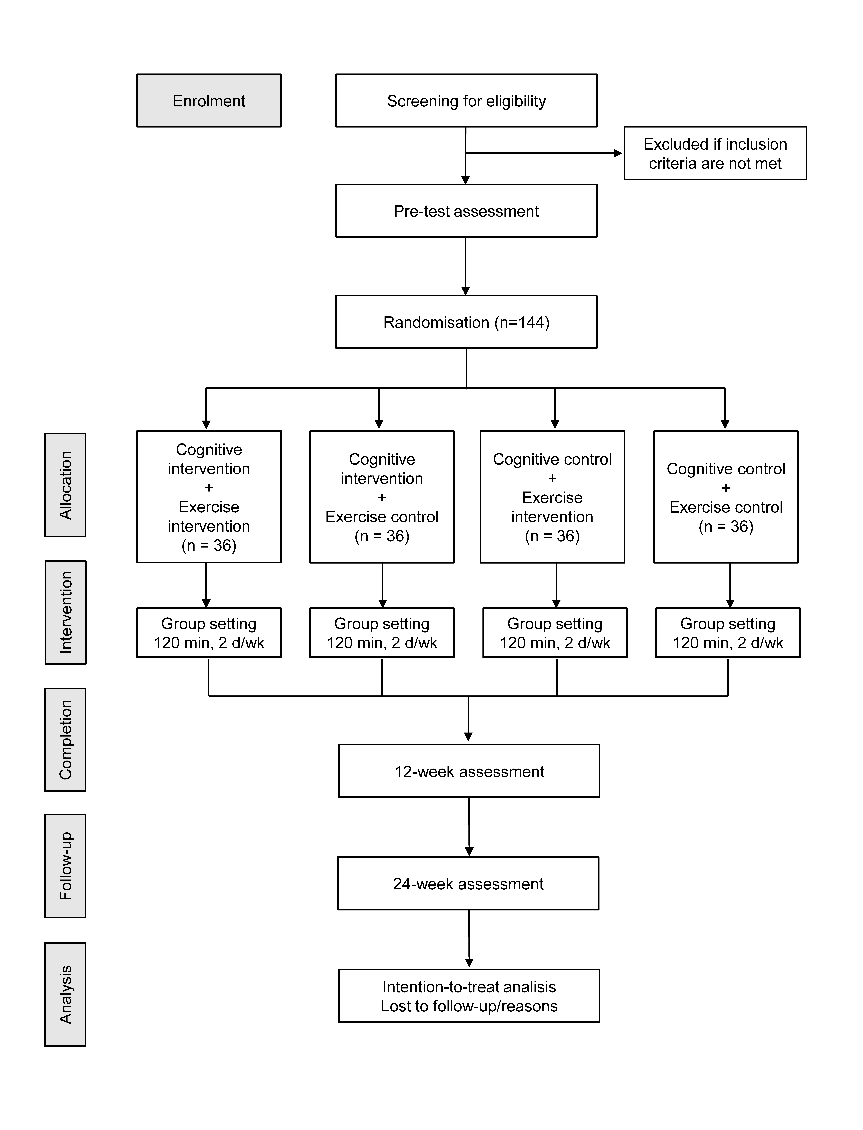   1. Adverse events   Even though this is a very low-risk trial, adverse events may happen during or after trial completion, such as medical illnesses or age-related declines in physical and cognitive functions. Participants will be closely monitored during the trial, however, accidents during physical exercise, or their way to the university site cannot be completely ruled out.   1. Outcome measures   No results available yet.   1. URL link to protocol file(s) with version and date   <https://clinicaltrials.gov/ct2/show/NCT03823183>   1. Brief summary   This RCT will investigate in older adults the synergetic effects of a simultaneous, group-based multidomain training program that combines cognitive video-game training with physical exercise, in comparison to those produced by cognitive training combined with physical control activity, physical training combined with cognitive control activity, or a combination of both control activities, and whether possible enhancements persist after a 3-month period without training. |
| 1. **IPD sharing statement** |
| Undecided. |
